# PdCo/Pd-Hexacyanocobaltate Hybrid Nanoflowers: Cyanogel-Bridged One-Pot Synthesis and Their Enhanced Catalytic Performance

**DOI:** 10.1038/srep32402

**Published:** 2016-08-30

**Authors:** Zhen-Yuan Liu, Geng-Tao Fu, Lu Zhang, Xiao-Yu Yang, Zhen-Qi Liu, Dong-Mei Sun, Lin Xu, Ya-Wen Tang

**Affiliations:** 1 Jiangsu Key Laboratory of New Power Batteries, Jiangsu Collaborative Innovation Centre of Biomedical Functional Materials, School of Chemistry and Materials Science, Nanjing Normal University, Nanjing 210023, PR China; 2 Materials Science and Engineering Program & Texas Materials Institute, the University of Texas at Austin, Austin, Texas 78712, United States; 3 Department of Applied Chemistry, Graduate School of Engineering, Hiroshima University, Hiroshima 739-8527, Japan

## Abstract

Elaborate architectural manipulation of nanohybrids with multi-components into controllable 3D hierarchical structures is of great significance for both fundamental scientific interest and realization of various functionalities, yet remains a great challenge because different materials with distinct physical/chemical properties could hardly be incorporated simultaneously into the synthesis process. Here, we develop a novel one-pot cyanogel-bridged synthetic approach for the generation of 3D flower-like metal/Prussian blue analogue nanohybrids, namely PdCo/Pd-hexacyanocobaltate for the first time. The judicious introduction of polyethylene glycol (PEG) and the formation of cyanogel are prerequisite for the successful fabrication of such fascinating hierarchical nanostructures. Due to the unique 3D hierarchical structure and the synergistic effect between hybrid components, the as-prepared hybrid nanoflowers exhibit a remarkable catalytic activity and durability toward the reduction of Rhodamine B (RhB) by NaBH_4_. We expect that the obtained hybrid nanoflowers may hold great promises in water remediation field and beyond. Furthermore, the facile synthetic strategy presented here for synthesizing functional hybrid materials can be extendable for the synthesis of various functional hybrid nanomaterials owing to its versatility and feasibility.

Rational hybridization and nanostructure engineering allow for achieving optimized or diversified material functionalities and thus have attracted increasing research interests in nanochemistry community^1^,^2^,^3^,^4^,^5^,^6^,^7^,^8^,^9^,^10^. Hybrid nanostructures with multi-components in one nanoscale entity could not only possess combined properties from the individual component, but also be capable of demonstrating new synergistic effects, which are induced by the nanoscale interactions and inaccessible from the isolated components or their physical mixtures. Therefore, a great number of nanocomposites have been synthesized and hold promising applications in various fields, including catalysis^11^,^12^,^13^, energy conversion and storage^14^,^15^,^16^,^17^, optoelectronic devices^18^,^19^,^20^, *etc*. Generally, the exceptional synergistic functionalities of the hybrid nanostructures are not only determined by the nature of each constituent component, but also more sensitively dependent upon the geometrical arrangement of the building units. Specifically, elaborate architectural manipulation of low dimensional (0D, 1D and 2D) primary building blocks into controllable 3D hierarchical structures is of great significance for both fundamental scientific interest and technological applications, and also provides a promising approach toward the future realization of functional nanodevices^5^,^10^. Owing to their unique structures, 3D hierarchical structures could possess the advantages of the pristine building blocks, and more importantly, also may exhibit even new physicochemical characteristics induced by coupling or ensemble effects, in comparison with their 1D or 2D counterparts^21^,^22^,^23^,^24^,^25^. Hitherto, despite considerable achievements have been made in such interesting field, it still remains a great challenge to develop a facile and controllable route for the construction of hierarchical architectures. Especially, it is extremely difficult to integrate multi-components into a hybrid hierarchical nanostructure based on the protocols established before, because different materials with distinct physical/chemical properties could hardly be incorporated simultaneously into the synthesis process. Therefore, it is highly desirable to develop a straightforward synthetic approach to generate nanohybrids with hierarchical architectures.

Cyanogel, pioneered by Bocarsly, is a kind of coordination polymer obtained from the reaction of aqueous solutions of a tetrachlorometalate ([RCl_4_]^2−^, R = Pd, Pt, Ir, Sn) and a transition metal cyanometalate ([M(CN)_n_]^2−/3−^, n = 4, 6; M = Co, Fe, Ru, Os, Ni, Cr), as illustrated in Equation (1) in Supplementary Information^26^,^27^,^28^,^29^. By taking advantages of the structural features of cyanogels, such as 3D characteristic backbones and uniform distribution of the two kinds of metal ions, we have developed a versatile cyanogel-based approach for the synthesis of various 3D noble metal-based nanostructures with improved catalytic performances^30^,^31^,^32^,^33^,^34^. Our previous results demonstrate that the cyanogel-based approach has the capacity to address some of the challenges in controlled construction of hybrid nanomaterials.

Herein, for the first time, we extend the capability of one-pot cyanogel-based hydrothermal approach to achieve 3D flower-like metal/Prussian blue analogue nanohybrids, namely PdCo/Pd-hexacyanocobaltate (PdCo/PdHCC), constructed by numerous radial 2D ultrathin nanosheets, by using K_2_PdCl_4_/K_3_Co(CN)_6_-PEG hybrid cyanogel as the reaction precursor (Fig. 1). Control experiments indicate that the elaborate co-existence of cyanogel and PEG is crucial for the generation of such interesting hierarchical architecture. Remarkably, due to the unique 3D hierarchical structure and the synergistic effect between hybrid components, the as-prepared PdCo/PdHCC hybrid nanoflowers exhibit an excellent catalytic activity and durability toward the reduction of Rhodamine B (RhB) by NaBH_4_, as compared with the Pd and PdHCC nanoparticles.

## Results and Discussion

### Physicochemical characterization of PdCo/PdHCC hybrid nanoflowers

For a standard synthesis of PdCo/PdHCC hybrid nanoflowers, yellowish jelly-like K_2_PdCl_4_/K_3_Co(CN)_6_-PEG hybrid cyanogel was firstly generated by mixing K_2_PdCl_4_-PEG solution and K_3_Co(CN)_6_-PEG solution. Upon a hydrothermal treatment, the hybrid cyanogel could be readily converted to 3D nanostructures owing to its intrinsic 3D characteristic backbones and the structural-directing effect of PEG. Simultaneously, PdCo alloy nanoparticles could be *in-situ* generated thanks to the weak reducing ability of PEG. Thus, the as-synthesized hybrid cyanogel could be evolved to uniform 3D flower-like PdCo/PdHCC nanohybrids after a hydrothermal treatment (see Experimental section for details).

**Figure 2:**
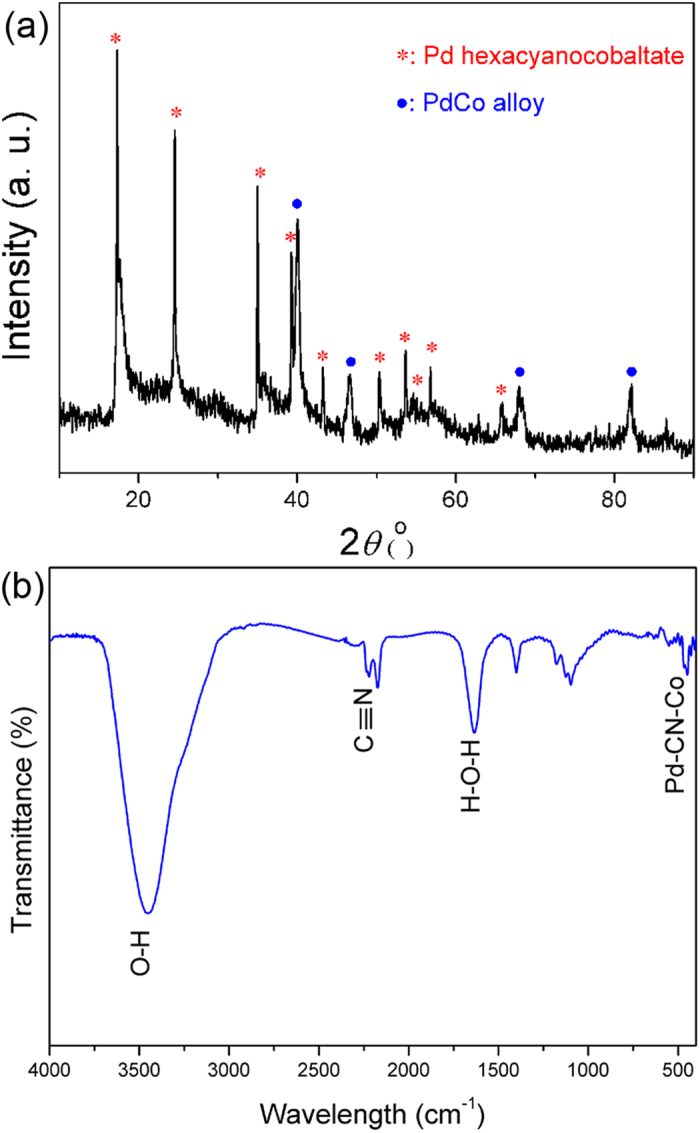
(**a**) XRD pattern and (**b**) FTIR spectrum of the synthesized PdCo/PdHCC hybrid hierarchical nanoflowers.

X-ray diffraction (XRD) pattern in Fig. 2a indicates that both face-centered cubic (*fcc*)-phased PdCo alloy and Prussian blue analogue, Pd-hexacyanocobaltate, coexist in the obtained product^35^. Figure S1 schematically illustrates the possible crystal structure of PdHCC. Analogous to Prussian blue, it has a three-dimensional cyano-bridged bimetallic basic unit with alternating Pd(II) and Co(III) located in a *fcc* lattice^36^,^37^,^38^. Fourier transform infrared (FTIR) analysis (Fig. 2b) shows the characteristic stretching peaks of C≡N around 2170 cm^−1^ and the absorption peak of Pd-CN-Co at 452 cm^−1^, confirming the successful formation of Prussian blue analogue^39^,^40^. The thermal stability of the product was investigated by thermogravimetry analysis (TGA) under air atmosphere. As displayed in Figure S2, the weight loss from room temperature to ~115 °C is caused by the loss of free water^41^. The weight loss in the temperature range of 195–240 °C can be assigned to the removal of coordinating water for Prussian blue^42^. When the temperature is increased above 245 °C, the PdHCC species begin to thermally decompose in air^40^.

**Figure 3:**
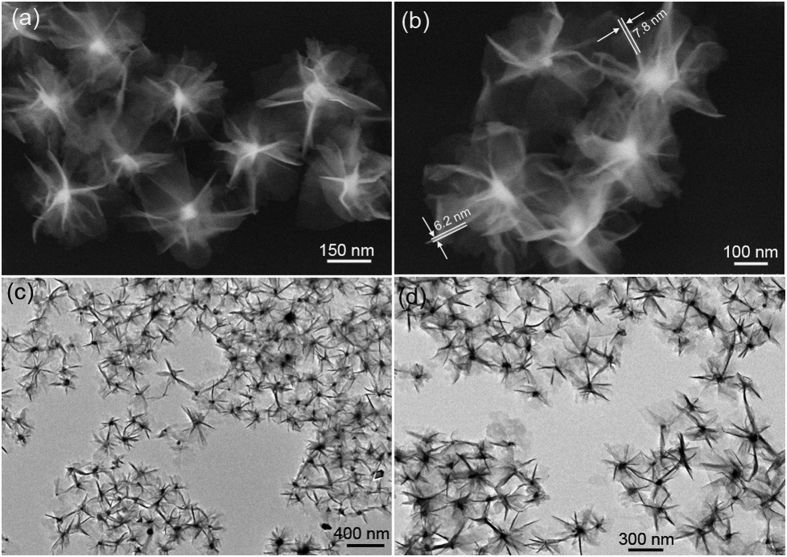
(**a,b**) Representative SEM images and (**c**,**d**) typical TEM images of the obtained PdCo/PdHCC hybrid hierarchical nanoflowers.

A panoramic scanning electron microscopy (SEM) image shown in Fig. 3a demonstrates that the product is almost entirely composed of uniform nanoflowers with diameter of 320 ± 20 nm. No other morphologies could be detected, indicating a high yield of these hierarchical structures. It is clearly shown that these nanoflowers are actually built from 2D ultrathin flexible nanosheets with an average thickness around 7 nm (Fig. 3b). These flexible nanosheets are eradiated from the central region to form open porous hierarchical structures, which may give rise to a large surface area and thus improved physicochemical properties. As shown in Fig. 3c,d, the typical transmission electron microscopy (TEM) images reveal that the as-synthesized sample exhibits urchin-like structures with an average diameter ~320 nm, which further confirms that the sample is constructed by radial nanosheets, in good agreement with the SEM observation. These nanoflowers could maintain their integrity upon sonication treatment for 30 min, suggesting the existence of strong chemical bonds between the building blocks.

**Figure 4:**
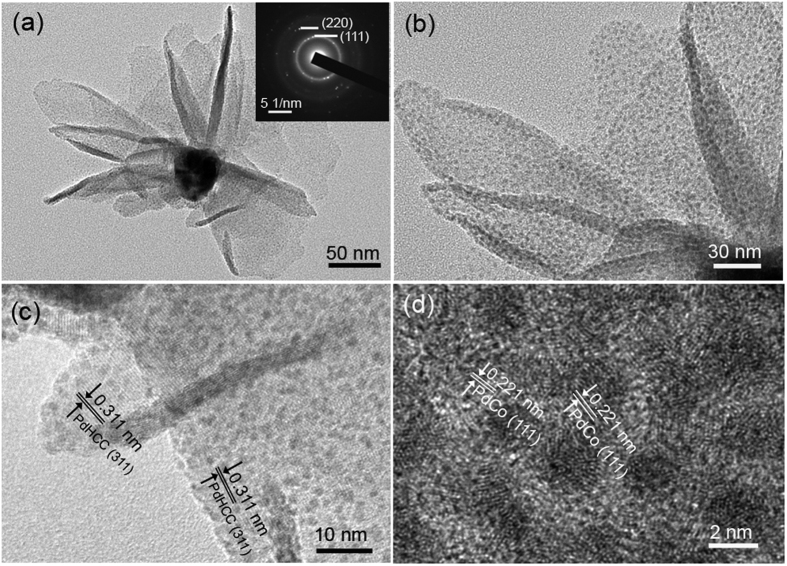
(**a**) TEM image of an individual PdCo/PdHCC hybrid nanoflower, (**b**) Magnified TEM of the “petals” from the nanoflower, and (**c**,**d**) HRTEM images performed on the petal.

From the TEM image of an individual nanoflower (Fig. 4a), it is obvious that the “petals” tend to bend and curl, reflecting the flexibility of the building blocks. The corresponding selected area electron diffraction (SAED) pattern (inset of Fig. 4a) implies a polycrystalline nature of the nanoflower and the diffraction dots are well consistent with the (111) and (220) planes of *fcc*-structured alloy phase. Magnified TEM image in Fig. 4b vividly reveals that uniform ultrafine nanoparticles are highly dispersed on the surface of the nanosheets. Figure 4c and d display the high-resolution TEM (HRTEM) images of the nanosheets and the homogeneously dispersed nanoparticles, respectively. The fringe spacing of 0.311 nm observed from the petal can be indexed to the (311) planes of PdHCC, while lattice fringes of 0.221 nm in nanoparticles can be attributed to the (111) planes of *fcc*-phased PdCo alloy. Notably, the measured lattice fringes of (111) planes in the nanoparticles are smaller than that of the pure Pd (0.225 nm, JCPDS 46-1043). Such shrinkage of the lattice fringe further verifies the formation of PdCo alloyed nanoparticles. Consistent with the SEM and TEM observations, high-angle annular dark-field scanning TEM (HAADF-STEM) shown in Figure S3 verifies that the as-prepared nanoflowers are built from 2D nanosheets. The elemental mapping further reveals the presence and uniform distributions of Pd and Co throughout the hybrid nanoflowers.

**Figure 5:**
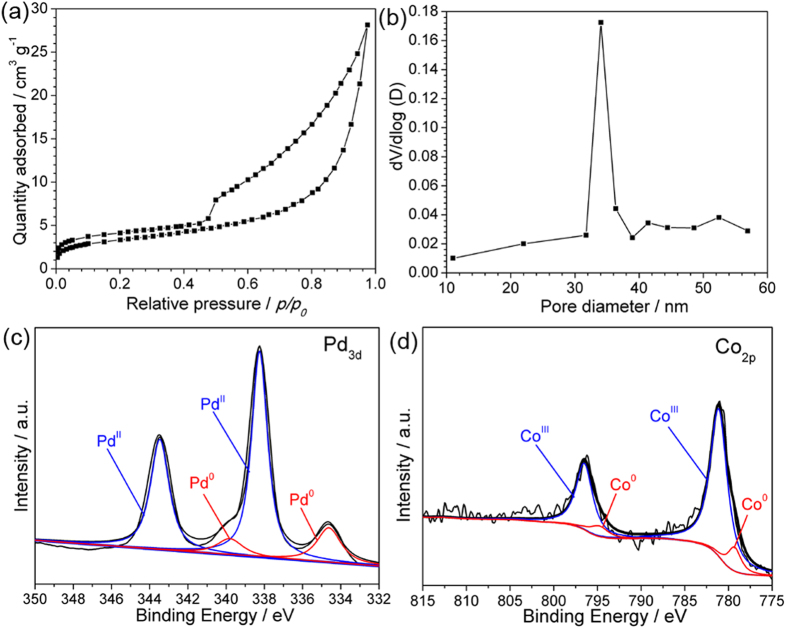
(**a**) N_2_ adsorption-desorption isotherms and (**b**) pore-size distribution curve of the PdCo/PdHCC nanoflowers. (**c**,**d**) XPS spectra of the Pd 3d and Co 2p regions for the PdCo/PdHCC nanoflowers.

The porosity and Brunauer-Emmett-Teller (BET) surface area of the as-synthesized PdCo/PdHCC nanoflowers were investigated through N_2_ adsorption-desorption measurements. As displayed in Fig. 5a, the N_2_ adsorption-desorption isotherms of PdCo/PdHCC nanoflowers can be categorized as type IV with a significant hysteresis loop observed in the relative pressure (*p*/*p*_0_) range of 0.5–1.0, which implies the presence of *meso*-pores (2–50 nm in size)^43^. This result can be further confirmed by corresponding pore-size distribution curve (Fig. 5b), in which a peak centred at 34 nm can be observed. As revealed by the SEM observation, these *meso*-pores can be attributed to the space between the intercrossed 2D nanosheets^44^. The BET surface area of the PdCo/PdHCC nanoflowers calculated from N_2_ isotherms is 32.8 m^2^ g^−1^. The valance states of Pd and Co in the hybrid nanoflowers were examined by X-ray photoelectron spectroscopy (XPS) technique, revealing that both metallic and oxidic states of Pd and Co exist in the hybrid nanoflowers (Fig. 5c,d)^45^,^46^. These results further verify the hybrid compositions as PdCo/Pd hexacyanocobaltate.

To develop an understanding of the mechanism behind the formation of PdCo/PdHCC nanoflowers, the chemical fate of each involved reagent has been considered. When PEG is absent from the reaction system while the other reaction parameters remain unchanged, although the yellowish jelly-like cyanogel could be still formed (Figure S4a), the resulting product achieved after the hydrothermal treatment is made of the isolated palladium hexacyanocobaltate nanoparticles with an average size of 70 nm, as confirmed by XRD and TEM images (Figure S4b–d). When there is no K_3_Co(CN)_6_ introduced, the reduction of K_2_PdCl_4_ by PEG could only produce irregular aggregated nanoparticles (Figure S5a). In comparison, the hydrothermal treatment of the mixture only containing K_3_Co(CN)_6_ and PEG could generate intercrossed nanochains (Figure S5b). Collectively, all these results unambiguously suggest that the presence of PEG and the formation of cyanogel are indispensable for the successful formation of 3D PdCo/PdHCC hybrid nanoflowers.

**Figure 6:**
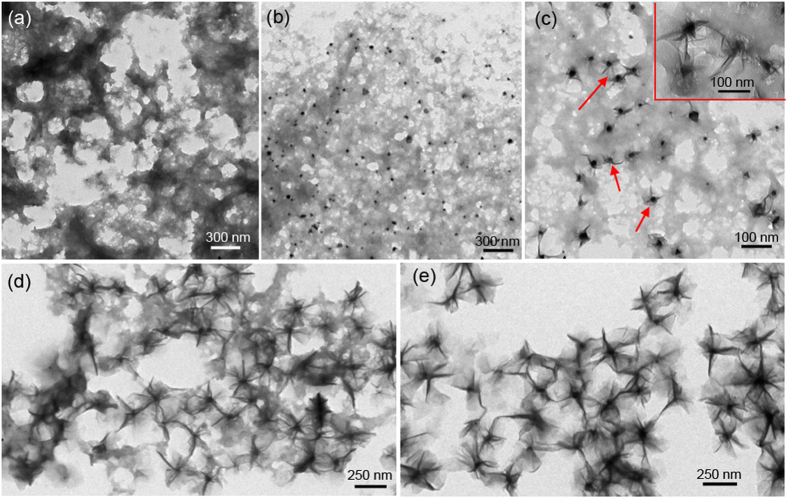
TEM images of the PdCo/PdHCC nanoflower intermediates collected at different reaction intervals. (**a**) 1 h, (**b**) 2 h, (**c**) 3 h, (**d**) 4 h and (**e**) 5 h.

Furthermore, time-dependent experiments have been carefully carried out to reveal the morphological evolution. Figure 6 illustrates the representative TEM images of the intermediate products collected at different reaction intervals. As shown in Fig. 6a, the sample consists of numerous flocculated agglomerates without a discernible morphology when the hybrid cyanogel was hydrothermally treated for 1 h. As the reaction time was prolonged to 2 h, the flocculation tended to aggregates together, forming a large number of nanoparticles (Fig. 6b). Interestingly, some nanosheets began to germinate from the surface of nanoparticles when the reaction time was increased to 3 h, as indicated by red arrows and inset of Fig. 6c. As a consequence of continuous growth, development and ripening, more and more nanosheets sprouted from the surface of nanoparticles and the obtained hierarchical architectures became ripening and plumy, accompanied by the gradual depletion of the flocculation (Fig. 6d). Eventually, uniform well-developed 3D PdCo/PdHCC hybrid nanoflowers constructed by 2D nanosheets were formed when the reaction time was proceeded more than 5 h (Fig. 6e).

**Figure 7:**
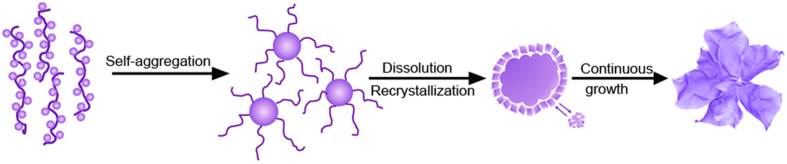
Schematic illustration of the proposed formation mechanism of PdCo/PdHCC hierarchical nanoflowers.

Based on the above TEM observations, the possible formation mechanism of the 3D PdCo/PdHCC hybrid nanoflowers could be proposed as follows. As we know, PEG is a kind of nonionic surfactant which possesses hydrophilic -O- and hydrophobic -CH_2_-CH_2_- radicals on its long chains, and usually serves as structure-directing agent or soft template for engineering ordered nanostructures due to its selective adsorption to inhibit crystal growth and thus modify the morphology of nanocrystallite^47^,^48^. In this work, PdCo-based cyanogel will be enwrapped into the coil of intertwisted PEG and form flocculated agglomerates when the precursors are initially mixed. From the thermodynamic viewpoint, the flocculation has a tendency to self-aggregate into nanoparticles to minimize the total surface energy when hydrothermally treated. As the reaction proceeds, the formed nanoparticles continue to grow by combining with the remaining flocculated agglomerates and recrystallize. Meanwhile, PEG may selectively bind to certain specific crystallographic facets^49^. Such a preferential adsorption could effectively facilitate the anisotropic growth, leading to the formation of 2D nanosheets. Therefore, with the further increase of reaction time, more and more 2D nanosheets are germinated from the surface of nanoparticles, and the nanoparticles gradually evolve into hierarchical nanoflowers at a later stage. Therefore, the formation of 3D PdCo/PdHCC hybrid nanoflowers can be rationally expressed as a “nucleation-aggregation-dissolution-recrystallization” mechanism^50^,^51^. During the formation of PdHCC nanoflowers with the assistance of PEG, the PdHCC could be partially reduced by PEG to form PdCo alloy nanoparticles which are simultaneously dispersed on the surface of PdHCC nanoflowers. The plausible formation process can be schematically illustrated in Fig. 7.

### Catalysis for the hydrogenation of RhB

**Figure 8:**
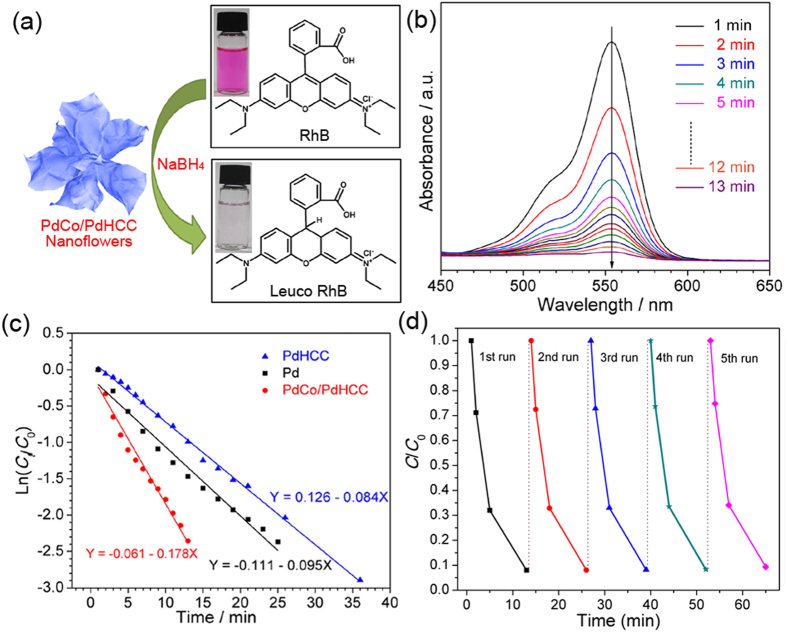
(**a**) Schematic illustration of the catalytic reduction of RhB with NaBH_4_ in the presence of PdCo/PdHCC nanoflowers. (**b**) UV-vis absorption spectra of RhB during the reduction catalyzed by PdCo/PdHCC nanoflowers. (**c**) First-order kinetics, ln(*C*/*C*_0_) *vs t*, for the catalytic hydrogenation of RhB solution via nanocatalysts in the presence of NaBH_4_. (**d**) Cycling test of hydrogenation of RhB over 5 cycles by using PdCo/PdHCC hybrid nanoflowers.

Such a hierarchical architecture and integrated multiple compositions in nanoscale might bring out some unusual physiochemical properties. As a proof-of-concept application of this intriguing hybrid nanostructure, the obtained 3D PdCo/PdHCC hybrid nanoflowers were employed as a catalyst for the hydrogenation of RhB in the presence of NaBH_4_. The catalytic reduction of RhB is schematically illustrated in Fig. 8a 52. The characteristic absorption peak of RhB at 554 nm was selected to monitor the catalytic reduction process. For comparison, a series of control experiments were also performed under different conditions: (1) without NaBH_4_ but in the presence of PdCo/PdHCC hybrid nanoflowers, (2) without any catalyst but in the presence of excess NaBH_4_, and (3) catalyzed by Pd or PdHCC nanoparticles. As shown in Figure S6a, the physical adsorption experiment demonstrates that the PdCo/PdHCC hybrid nanoflowers have a very weak adsorption capability toward RhB (only 2.2% in 24 h), precluding the physical adsorption of RhB by PdCo/PdHCC hybrid nanoflowers. The further control experiment (Figure S6b) indicates that RhB is slightly reduced (3.6% in 60 min) in the presence of excess NaBH_4_ but without any catalyst. Whereas, upon the introduction of PdCo/PdHCC hybrid nanoflowers into the reaction system, the color of RhB solution changed from pink to colorless rapidly. As displayed in Fig. 8b, the maximal absorption of the RhB dye decreased significantly as reaction time went on, and the reduction reaction completed in 13 min, indicating the excellent catalytic performance of the PdCo/PdHCC hybrid nanoflowers. No deactivation or poisoning of the catalyst could be observed during the reaction. Although PdHCC or Pd nanoparticles could also catalyze the reduction of RhB, the periods for the complete reaction of the two reference materials are much longer as compared with the case of PdCo/PdHCC hybrid nanoflowers (36 or 25 min *vs*. 13 min), revealing their much lower reaction rates (Figure S6c,d).

As suggested by the previous studies, the hydrogenation reduction of RhB obeys a pseudo-first order kinetic law^53^. On the basis of the pseudo-first order kinetics, ln(*C*/*C*_0_) = *kt*, where *C* is the concentration of the RhB at time *t*, *C*_0_ is the initial concentration of the RhB solution, and the slope *k* is the apparent reaction rate, the ln(*C*/*C*_0_) is linearly dependent on the reaction time *t*. As shown in Fig. 8c, the calculated rate constant *k* with PdCo/PdHCC hybrid nanoflowers is 0.178 min^−1^, which is obviously larger than that in Pd (0.095 min^−1^) or PdHCC (0.084 min^−1^) case. The long-term stability of the prepared PdCo/PdHCC hybrid nanoflower sample was also evaluated through a cycling test. After each cycle of the 13-min test, the sample was washed and reused for reduction of RhB. As shown in Fig. 8d, no obvious decrease of catalytic activity was observed after five cycles, suggesting very high stability and long lifetime of PdCo/PdHCC hybrid nanoflowers during the hydrogenation process. As revealed by TEM and SEM images shown in Figure S7a,b, the 3D hierarchical flower-like structures could be well preserved without notable aggregation or detachment after five cycles. Moreover, as implied by the XPS results in Fig. 5c,d and Figure S7c,d, the valence states of Pd and Co in the hybrid nanoflowers almost kept consistent before and after cycling tests. All these results strongly manifest the excellent robustness of the 3D hierarchical nanoflowers.

Generally, the catalytic hydrogenation mechanism of RhB *via* noble metal-based nanocatalysts in the presence of NaBH_4_ could be explained as follows. The dye of RhB is electrophilic while BH_4_^−^ is nucleophilic as compared with the catalyst, demonstrating that the nucleophilic BH_4_^−^ can donate electrons to the catalyst, from where electrophilic dyes would capture electrons. So the catalyst serves as an electron relay for catalytic reduction of dyes in the presence of NaBH_4_^54^,^55^. In the present study, the high dispersity of PdCo nanoparticles with ultrafine size in PdCo/PdHCC nanoflowers not only helps to provide more catalytic sites, but also could effectively prevent the agglomeration of PdCo nanoparticles during the reaction. Furthermore, the hierarchical nanoflowers offer a high surface-to-volume ratio and have plenty of open *meso*-pores, providing more molecular accessibility, efficient transport paths and thus improved catalytic activity toward the reduction of RhB^56^,^57^,^58^.

In summary, we have developed a novel cyanogel-bridged one-pot synthesis approach for the generation of 3D flower-like metal/Prussian blue analogue nanohybrid, namely PdCo/Pd-hexacyanocobaltate, for the first time. The judicious introduction of PEG and the formation of cyanogel are indispensable for the successful formation of such fascinating hierarchical nanostructures. Owing to the unique 3D hierarchical structure and the synergistic effect between hybrid components, the as-synthesized hybrid nanoflowers exhibit an excellent catalytic activity and durability toward the reduction of RhB by NaBH_4_, which indicates that the hybrid nanoflowers may hold great promise in water remediation field and beyond, such as electrocatalysis and sensor, *etc*. Furthermore, the novel method developed in this work for synthesizing functional hybrid materials with hierarchical structures can be extended to the fabrication of various functional hybrid nanomaterials thanks to its versatility and feasibility.

## Methods

### Synthesis of PdCo/PdHCC hybrid nanoflowers

In a typical synthesis, 2.0 mL of 50 mM K_2_PdCl_4_ solution containing 340 mg PEG and 1.0 mL of 50 mM K_3_Co(CN)_6_ solution containing 170 mg PEG were mixed and kept still for 2 h at 30 °C, allowing for the formation of yellow jelly-like K_2_PdCl_4_/K_3_Co(CN)_6_-PEG hybrid cyanogel. Subsequently, the obtained cyanogel was transferred to a 20 mL Teflon-lined stainless autoclave and heated at 150 °C for 6 h. After being cooled to room temperature, the black product was separated by centrifugation, washed with 0.1 M HClO_4_ solution and water several times, and then dried at 40 °C in a vacuum oven for 12** **h. The acid-wash process could ensure the removal of possible byproducts or impurities. For comparison, the single-component Pd nanoparticles were prepared by only using K_2_PdCl_4_ as reaction precursor under the similar experimental conditions. The PdHCC nanoparticles were also prepared using the mixture of K_2_PdCl_4_ and K_3_Co(CN)_6_ yet without PEG as reaction precursors under the identical experimental conditions.

### Characterization

The morphology and particle size of the samples were investigated using a JEOL JEM-2010 transmission electron microscopy (TEM) operated at an accelerating potential of 200 kV. Scanning electron microscopy (SEM) images were captured on a Hitachi S-4800 scanning electron microscope, operating at 5 kV. X-ray diffraction (XRD) patterns were performed on Model D/max-rC X-ray diffractometer using Cu K*α* radiation source (λ = 1.5406 Å) and operating at 40 kV and 100 mA. X-ray photoelectron spectroscopy (XPS) measurements were carried out on a Thermo VG Scientific ESCALAB 250 spectrometer with a monochromatic Al K*α* X-ray source (1486.6 eV photons). The binding energy was calibrated with respect to C1s at 284.6 eV. The compositions of the catalysts were determined using the energy dispersive X-ray (EDX) technique. The Brunauer-Emmett-Teller (BET) specific surface area and pore size distribution were measured at 77 K using a Micromeritics ASAP 2050 system. Fourier transform infrared (FTIR) spectrum was recorded with a Nicolet 520 SXFTIR spectrometer. The UV-vis spectra were recorded at room temperature on a UV3600 spectrophotometer. Thermal analysis was performed on a Perkin Elmer thermogravimetric analyzer under air atmosphere with a heating rate of 10 °C min^−1^.

### Catalytic measurements

The reduction of organic dye molecules, such as RhB, with NaBH_4_ was chosen as a model reaction to evaluate the catalytic performance of the as-obtained PdCo/PdHCC nanoflowers. A NaBH_4_ solution (0.20 mg/mL) was freshly prepared and stored in refrigerator in the dark. The reduction of the RhB dyes was carried out in a quartz cuvette having a path length of 1 cm. For a catalytic reaction, 2 mL of 3.33 × 10^−5^ M RhB dye solution was mixed with 0.5 mL of 0.20 mg/mL NaBH_4_, followed by gentle shaking. Subsequently, 0.5 mL of 0.40 mg/mL PdCo/PdHCC nanoflower solution was added, and the progress of the reduction was monitored spectrophotometrically using an *in-situ* UV-vis spectrophotometer. For comparison, the catalytic processes catalyzed by PdHCC and monometallic Pd nanoparticles were also performed under the identical conditions.

## Additional Information

**How to cite this article**: Liu, Z.-Y. *et al.* PdCo/Pd-Hexacyanocobaltate Hybrid Nanoflowers: Cyanogel-Bridged One-Pot Synthesis and Their Enhanced Catalytic Performance. *Sci. Rep.* **6**, 32402; doi: 10.1038/srep32402 (2016).

## Supplementary Material

Supplementary Information

## Figures and Tables

**Figure 1:**
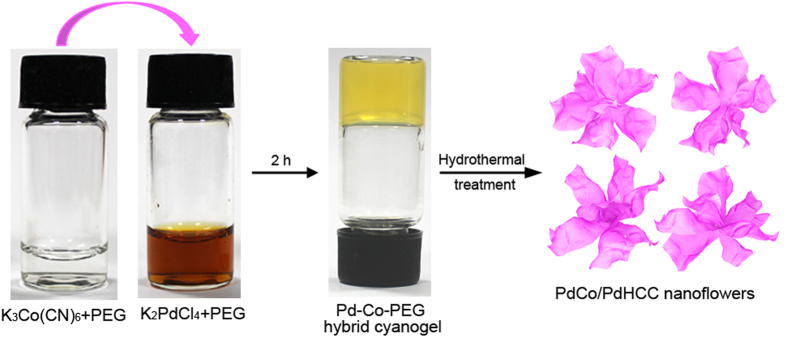
Schematic illustration of the formation of PdCo/PdHCC hierarchical nanoflowers using hybrid cyanogel as precursors.
