# Supplementary material for: PdCo/Pd-Hexacyanocobaltate Hybrid Nanoflowers: Cyanogel-Bridged One-Pot Synthesis and Their Enhanced Catalytic Performance
Source: Sci Rep. 2016 Aug 30;6:32402. doi: 10.1038/srep32402 (PMC5004103; doi:10.1038/srep32402)
Supplement: Supplementary Information [file srep32402-s1.doc]

**Supplementary Information**

**PdCo/Pd-Hexacyanocobaltate Hybrid Nanoflowers: Cyanogel-Bridged One-Pot Synthesis and Their Enhanced Catalytic Performance**

Zhen-Yuan Liu^1^, Geng-Tao Fu^1,3^, Lu Zhang^2^, Xiao-Yu Yang^1^, Zhen-Qi Liu^1^, Dong-Mei Sun^1^, Lin Xu*^1^, & Ya-Wen Tang*^1^

^1^ Jiangsu Key Laboratory of New Power Batteries, Jiangsu Collaborative Innovation Centre of Biomedical Functional Materials, School of Chemistry and Materials Science, Nanjing Normal University, Nanjing 210023, PR China; ^2^ Department of Applied Chemistry, Graduate School of Engineering, Hiroshima University, Hiroshima 739-8527, Japan; ^3^ Materials Science and Engineering Program & Texas Materials Institute, the University of Texas at Austin, Austin, Texas 78712, United States.

*Corresponding authors. Tel.: +86–25–85891651; fax: +86–25–83243286.

*E-mail address*: njuxulin@gmail.com (L. Xu); tangyawen@njnu.edu.cn (Y. W. Tang)

Equation (1). Formation equation of cyanogel.


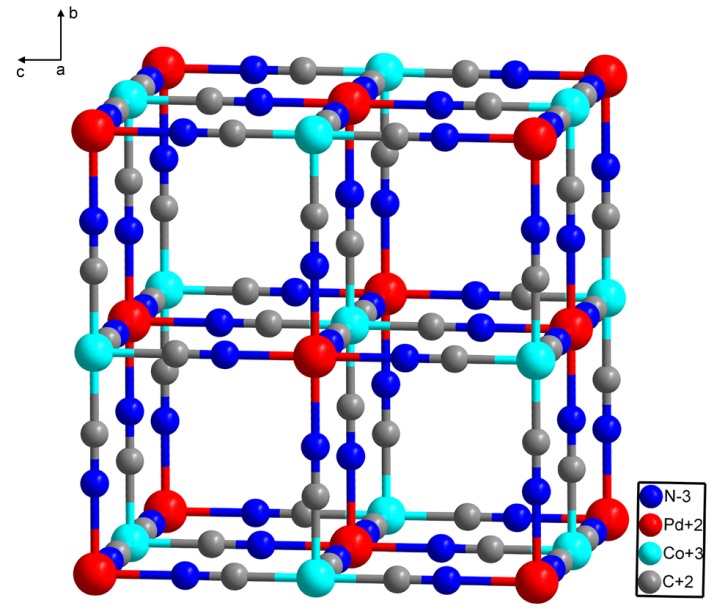


Figure S1. Crystal structure of palladium hexacyanocobaltate.


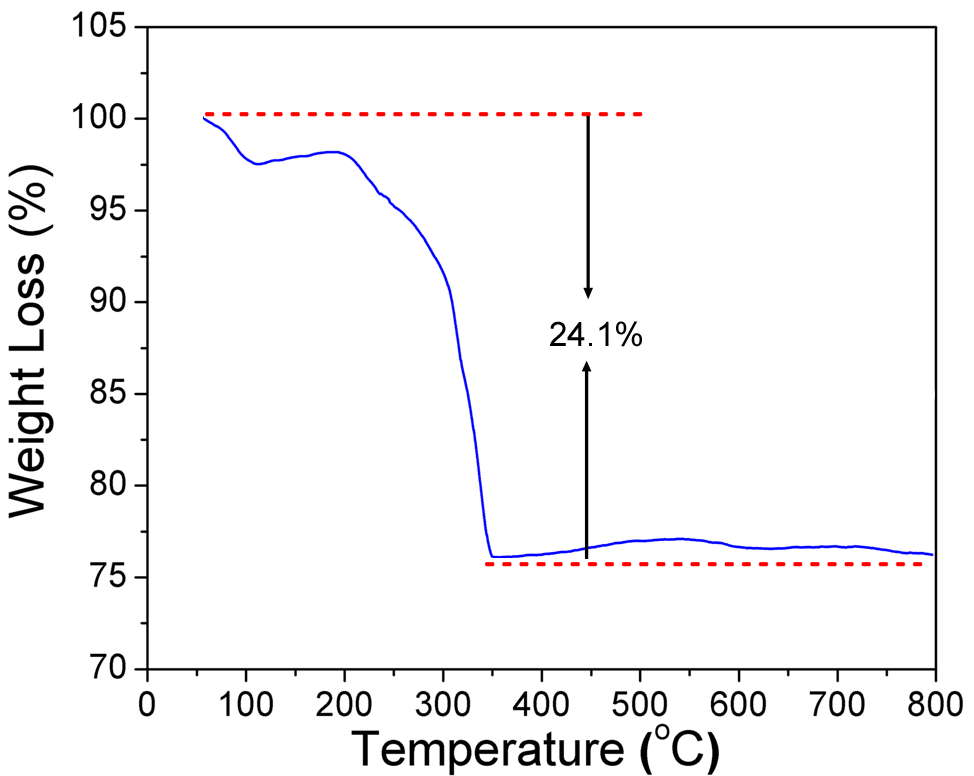


Figure S2. TGA curve of the synthesized PdCo/PdHCC hybrid nanoflowers.


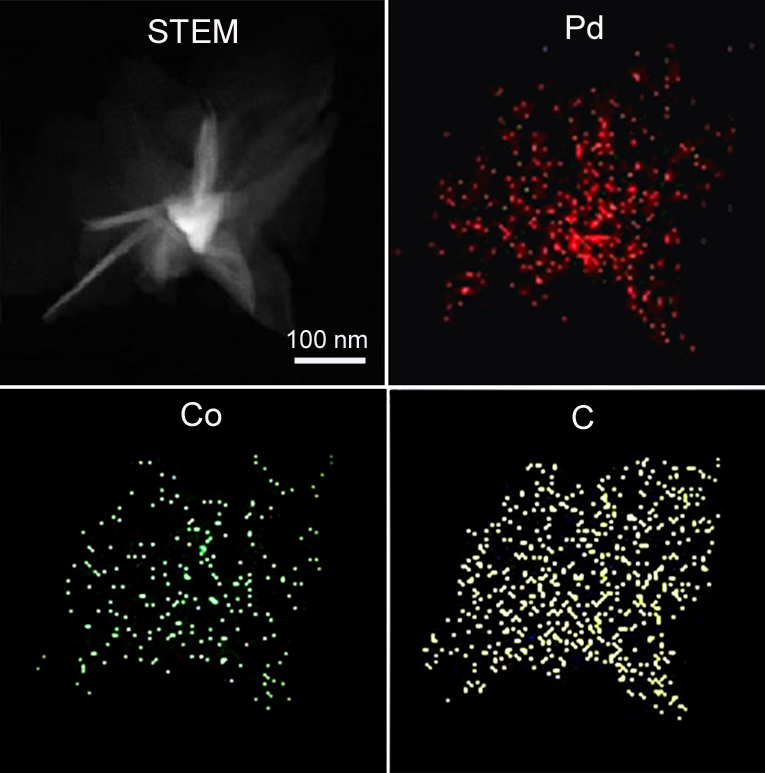


Figure S3. STEM image and elemental mapping of the prepared PdCo/PdHCC nanoflowers.


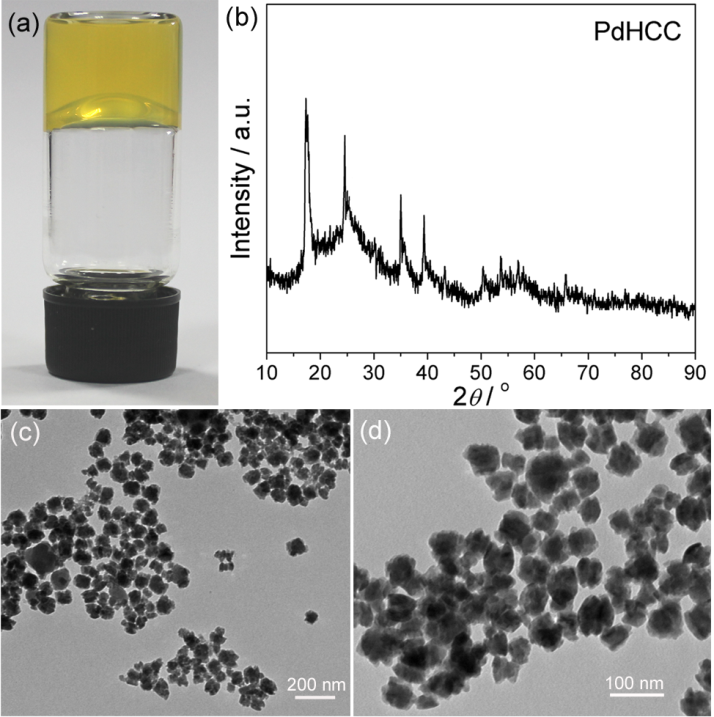


Figure S4. (a) Digital photograph of the as-obtained cyanogel in the absence of PEG. (b) XRD pattern of the PdHCC obtained without the assistance of PEG. (c)-(d) TEM images of the synthesized PdHCC nanoparticles.


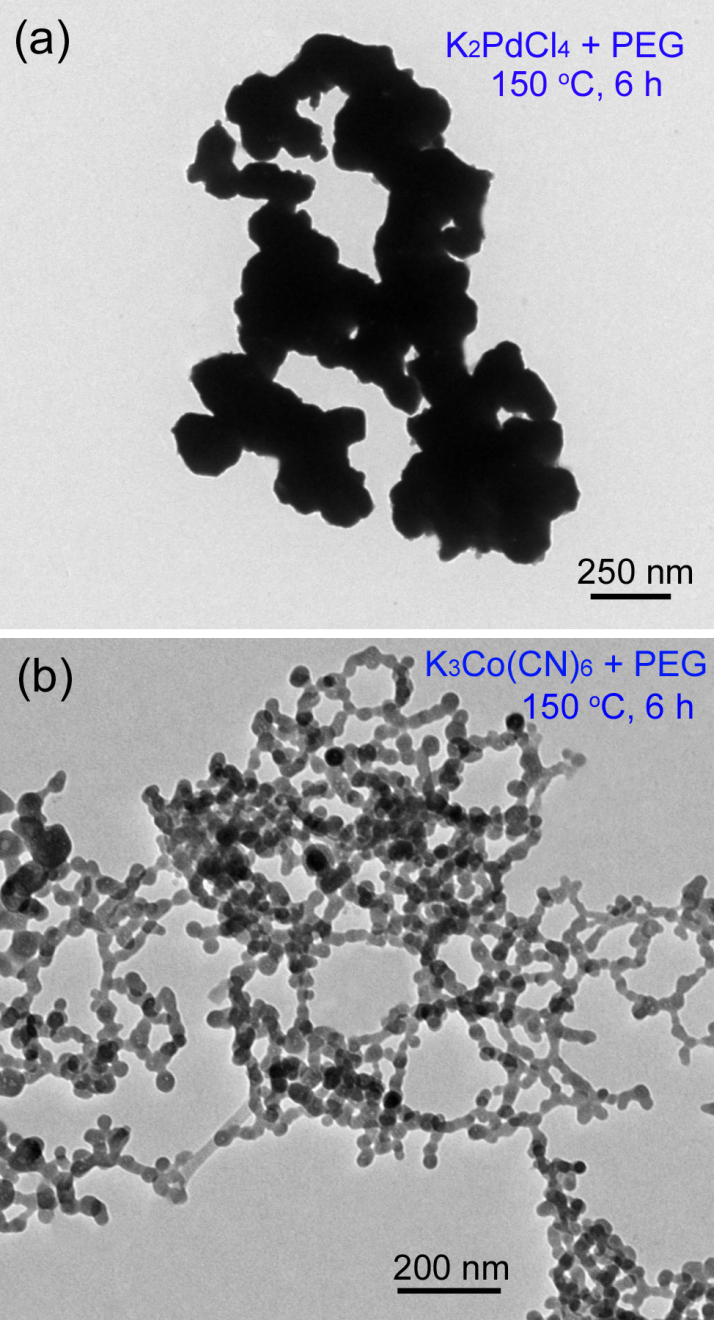


Figure S5. (a) TEM image of the product obtained from the reaction between K_2_PdCl_4_ and PEG, and (b) TEM image of the product obtained from the reaction between K_3_Co(CN)_6_ and PEG.


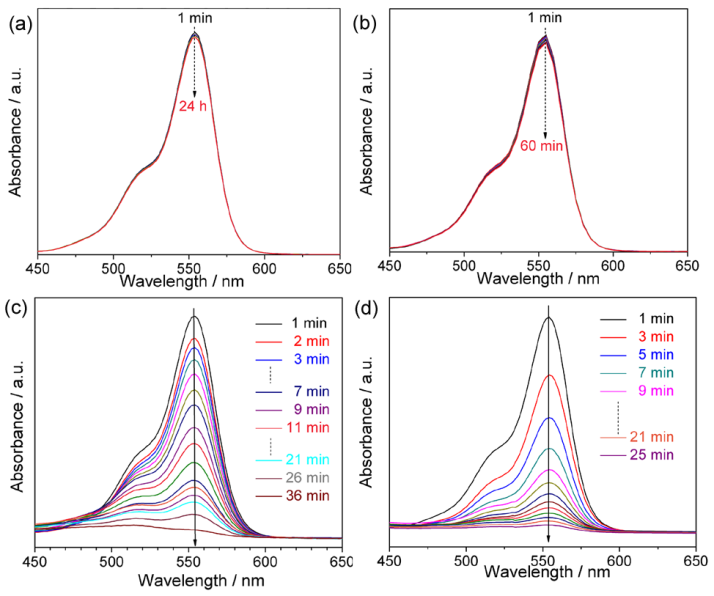


Figure S6. (a) UV-vis spectra of the RhB solution only in the presence of PdCo/PdHCC nanoflowers for 24 h. (b) UV-vis spectra of the RhB solution only in the presence of excessive NaBH_4_ for 1 h. (c) UV-vis spectra for the successive reduction of RhB with NaBH_4_ using PdHCC as catalyst. (d) UV-vis spectra for the successive reduction of RhB with NaBH_4_ using Pd nanoparticles as catalyst.


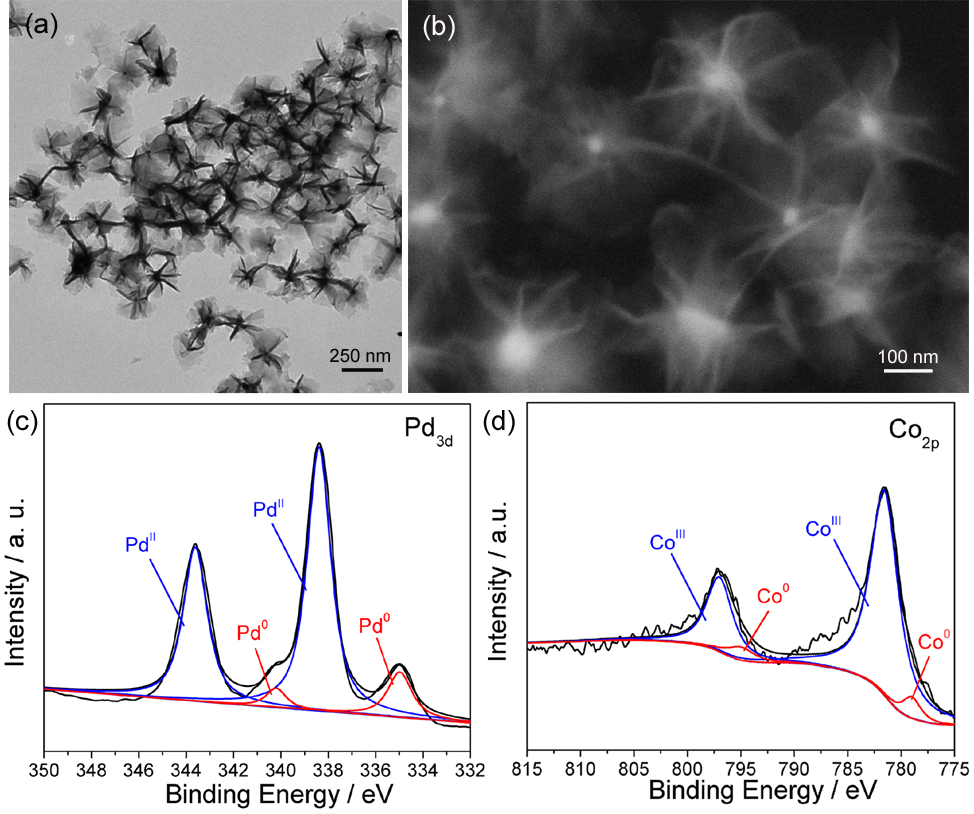


Figure S7. Morphology and surface chemistry characterization of the hybrid nanoflowers after cycling tests. (a) Typical TEM image, (b) SEM image, and (c)-(d) XPS spectra.
